# Perpetrator gender in adolescent sexual harassment: prevalence and mental health associations

**DOI:** 10.1007/s00787-025-02956-7

**Published:** 2026-02-04

**Authors:** Hilde Slaatten, Bente Storm Mowatt Haugland

**Affiliations:** 1 Regional Centre for Child and Youth Mental Health and Child Welfare, NORCE Norwegian Research Centre, Bergen, P.O. Box 7810, NO-5020 Norway; 2 Department of Clinical Psychology, Faculty of Psychology, University of Bergen, Bergen, Norway

**Keywords:** Sexual harassment, Adolescents, Depressive symptoms, Anxiety symptoms, Gender differences

## Abstract

Sexual harassment is widespread among adolescents and linked to adverse mental health outcomes. However, little attention has been paid to the gender of the perpetrator and its potential influence on the mental health of the victims. This study examines how the perpetrator’s gender relates to the prevalence and mental health correlates of sexual harassment among Norwegian adolescents aged 13 to 15. Cross-sectional data from 1,988 pupils in 40 secondary schools were used to assess boys’ and girls’ experiences of sexual harassment by both genders and to examine associations with depressive and anxiety symptoms. Overall, boys were more frequently identified as perpetrators, while girls were more often victims. Harassment by boys was associated with higher levels of depressive and anxiety symptoms in both girls and boys. In contrast, harassment by girls was associated with depressive and anxiety symptoms only in girls. The findings highlight the importance of considering perpetrator gender when assessing the impact of peer sexual harassment. Greater attention should be given to the experiences of young adolescents, who appear vulnerable to male-perpetrated harassment in particular. Targeted measures are needed to reduce the mental health burden of sexual harassment, and clinicians should consider these experiences in the assessment and treatment of adolescent anxiety and depression.

## Background and prevalence

Sexual harassment is widespread among young adolescents [1–4] and has been linked to elevated symptoms of depression and anxiety [2, 5]. However, limited research has examined how the gender of the perpetrator influences the prevalence and psychological consequences of sexual harassment. Most studies record harassment without identifying the perpetrator’s gender, limiting insight into the power dynamics involved.

In Norwegian law, sexual harassment is defined as “any form of unwanted sexual attention that has the purpose or effect of being offensive, frightening, hostile, degrading, humiliating, or troublesome [6]”. Unlike EU law, which requires harm to a person’s dignity, Norwegian legislation emphasizes the unwanted nature of the act [7]. In contrast, U.S. law sets a higher threshold, defining sexual harassment as behaviour that is sufficiently severe, persistent, or pervasive to interfere with a student’s education [8]. Unwanted contact with intimate areas is classified as sexual assault in Norway [9] and is not included here. Following prior research [8], this study defines sexual harassment as unwanted sexual attention, including verbal comments, gestures, physical contact, or display of pictures or images (in person or digitally), that occur among peers. Homophobic behaviours (e.g., slurs such as “faggot”) are driven by distinct mechanisms, and are therefore considered as a separate form of harassment, excluded from the definition of sexual harassment used in this study.

Despite being addressed in both Norwegian legislation and school curricula [10–12], sexual harassment remains pervasive in schools. A recent national survey found that nearly one in four pupils aged 13–19 had experienced sexual or homonegative name-calling in the past year [1]. Qualitative studies show that harassment of girls by boys is often normalized and therefore rarely reported or challenged [13–15]. These findings underscore the need to examine the structural and relational factors that shape harassment in early adolescence.

### Why perpetrator gender matters

The gender of the perpetrator is an important but understudied dimension of adolescent sexual harassment. Studies reporting both victim and perpetrator gender consistently find that boys are most often identified as perpetrators, regardless of the victim’s gender [16–18]. Some studies show more complex patterns; for example, heterosexual boys may report more harassment from girls than from boys, while girls most often report boys as perpetrators [19]. Across Western countries, girls are generally more likely than boys to experience sexual harassment [3, 5, 8, 20–24], although some studies find no significant gender differences [2, 4] or even higher rates among boys [4, 25]. Such discrepancies may partly reflect methodological variation, as studies including homonegative name-calling in their measurement of sexual harassment may overestimate male victimization [2, 4, 17]. Regarding self-reported perpetration, boys are consistently more likely than girls to report engaging in sexual harassment [5, 8, 17, 25], while girls seem to express stronger negative evaluations of such behaviour [26]. Taken together, these findings highlight the importance of considering perpetrator gender to better understand not only who is targeted but also how harassment is perceived, enacted, and experienced.

### Mental health implications

Perpetrator gender may also influence the psychological impact of sexual harassment [27]. Harassment by boys may be especially harmful perhaps because it reflects broader gendered power structures and social dominance [28–30]. Several studies have found stronger associations between sexual harassment and depressive or anxiety symptoms among girls than among boys [23, 31–33]. However, few studies have differentiated effects by perpetrator gender. One exception showed that being harassed by a boy, but not by a girl, was associated with elevated depressive and anxiety symptoms in both girls and boys [27]. By contrast, an unpublished Swedish study found no differences in discomfort by perpetrator gender [34]. Some research suggests that only certain types of harassment predict negative outcomes for girls, whereas boys appear less affected regardless of type [20, 24, 29] while other studies, however, report no gender differences in mental health outcomes [2, 5, 35]. From a developmental perspective, sexual harassment often increases during puberty as mixed-gender interaction intensifies and adolescents begin exploring early expressions of sexuality [17, 18]. This framework tends to interpret cross-gender harassment, especially by boys, as immature or ‘unskilled’ sexual expression, and same-gender harassment among girls as relational aggression, such as attempts to exclude others or damage friendships [17, 18]. However, this approach overlooks social hierarchies and gendered power. Boys harassed by girls may not experience comparable status loss, whereas girls harassed by boys often face both emotional harm and reputational damage. Qualitative studies show that many girls feel expected to tolerate harassment, often responding with avoidance or minimization and self-blame when behaviour is not seen as “serious” [13, 14]. The emotional toll may be heightened if harassment is trivialized by peers or teachers, leading some girls to silence or resignation [13, 15]. In contrast, same-gender harassment among girls may function as relational conflict or social regulation, while for boys, harassment from other boys may undermine social status and challenge masculinity norms that are central to belonging in early adolescence.

### Knowledge gap and study aim

Despite increasing awareness of male-perpetrated harassment following the MeToo movement [36], the experiences of younger adolescents have received limited attention. Early adolescence is a formative period when gender roles and peer norms are negotiated, and experiencing sexual harassment at this stage may influence emotional and social development, identity, self-concept and mental health. Understanding how perpetrator gender shapes both the prevalence and the psychological impact of harassment is essential for informing prevention and early intervention in school and clinical settings.

This study examines (1) the prevalence of sexual harassment by boys versus girls and (2) how perpetrator gender relates to depressive and anxiety symptoms among Norwegian adolescents aged 12–14. Based on previous research, we hypothesized that (1) both girls and boys would report being more frequently harassed by boys than by girls, and (2) harassment by boys would be associated with higher levels of depressive and anxiety symptoms for both genders.

## Method

### Design

Data for this study was collected as part of the baseline assessment for a cluster randomized controlled trial evaluating a school-based intervention targeting sexual harassment [60]. Data collected prior to randomization and before any exposure to the intervention were included in the present analyses.

### Participants and procedures

In March–May 2019 and March–May 2020, all eligible Norwegian lower secondary schools (grades 8–9) in the counties of Rogaland, Vestland, Nordland, and Buskerud/Viken, as well as in the cities of Oslo and Trondheim, were invited to participate. Schools were invited by postal and email contact with follow-up phone calls. In total, 479 eligible schools across these counties and cities were contacted. Schools were eligible if Norwegian was the primary language of instruction, and they served a general student population. Schools primarily enrolling pupils with special educational needs were not invited.

Forty-one schools (8.6% of all contacted; including two private schools) agreed to participate and signed participation agreements. After approval, digital parental consent forms were distributed through each school’s home-school communication platform. Data collection was conducted electronically in participating schools between March and May of each year. Surveys were administered by teachers during school hours. Teachers provided survey links to pupils with parental consent, who completed the electronic questionnaire during regular class hours. Across the 41 schools, 5,405 pupils were eligible, and parental consent was obtained for 2,881 (53.3%) pupils. One school withdrew prior to the administration of the survey due to limited resources, leaving 40 schools in the analytic sample. Of the pupils with parental consent, 1,988 pupils completed the baseline questionnaire (36.8% of all eligible pupils). The main reason pupils with parental consent did not participate was that entire classes did not complete the survey as planned. The final sample comprised 950 boys (47.8%) and 1,038 girls (52.2%). The 1,988 participants were distributed across 40 lower secondary schools, with the number of participating pupils per school ranging from 4 to 147. Both urban and rural schools were represented, with 52.2% of participants attending schools located in municipalities defined as urban areas, indicating that the sample reflects typical variation in Norwegian school size and context. Participants were in grades 8–9 and aged 13–15 years. Fifty two pupils indicated a gender identity other than “boy” or “girl,” or reported being born with a different sex than the gender indicated in the initial question.

### Ethics

The study was approved by the Regional Committees for Medical and Health Research Ethics in Norway (REK: 231585).

### Measures

#### Sexual harassment victimization

Sexual harassment victimization was assessed using a 13-item questionnaire, introduced by the question: “How often have you experienced the following behaviours against your will during the last two months, conducted by a pupil at your school?”. Ten items were adapted from the American Association of University Women (AAUW) Survey on “Bullying, Teasing, and Sexual harassment in school” [37] (Table 1). To include digital harassment, an additional item was modified from the AAUW survey on “Sexual Harassment in School” survey [8]: “Sent unwelcome sexual comments or pictures, or had someone post them about or of you by mobile or online”. Items assessing sexual coercion and homophobic harassment were excluded, as these were considered distinct from sexual harassment. Two additional items were included from previous research studies to capture additional forms of sexual harassment: “Made comments about or rated the parts of your body that make you a boy or a girl” [17] and “Called you “whore” or similar words” [38].

**Table 1:** Sexual harassment victimization in the last two months reported by boys and girls

	BOYS		GIRLS		
	Harassed by boys	Harassed by girls	Harassed by boys	Harassed by girls	Total
Type of harassment	%	%	%	%	%
Made sexual comments, jokes, gestures, or looks	13.4	5.4	27.1	9.5	23.6
Showed, gave or left you sexual pictures, photographs, illustrations, messages, or notes	5.5	2.8	12.5	4.3	11.1
Wrote sexual messages/graffiti about you on bathroom walls, in locker rooms, etc.	1.2	1.5	2.0	1.8	2.6
Spread sexual rumors about you	5.8	4.1	14.3	8.9	12.2
Touched, grabbed, or pinched you in a sexual way	10.1	5.2	11.4	7.2	14.7
Intentionally brushed up against you in a sexual way	6.6	5.2	12.0	3.4	12.4
Pulled at your clothing in a sexual way	2.9	2.3	3.9	3.2	5.1
Pulled off or down your clothing	5.9	1.4	1.6	2.1	5.0
Blocked your way or cornered you in a sexual way	3.1	2.5	6.7	1.7	6.2
Forced you to kiss him/her	0.8	1.6	1.2	1.3	2.0
Sent unwelcome sexual comments, or pictures or had someone post them about or of you by mobile or online	2.1	2.1	5.9	3.7	5.7
Made comments about or rated the parts of your body that makes you a boy or a girl	6.5	3.5	18.7	7.7	15.2
Called you “whore” or similar words	11.5	5.1	31.2	14.5	25.7
OVERALL: reporting at least one episode of sexual harassment	29.2	14.7	48.5	28.8	43.6

All items were modified to focus specifically on harassment by school peers within the last two months. Response options for each item were: “I have not experienced this in the last two months”, “It has only happened once or twice”, “Two or three times a month”, “About once a week”, and “Several times a week”. Each item was followed by the question: “Who did this to you?”, with three response options: “One or several boys”, “One or several girls” and “One or several boys AND one or several girls”. Each item was dichotomized (0 = no, 1 = yes) based on whether the behaviour was reported at least once in the last two months. Two total scores were calculated by summing the number of different types of sexual harassment experienced by perpetrator gender (i.e., harassment by boys and harassment by girls), yielding possible scores from 0 to 13 for each subscale.

#### Depressive symptoms

Depressive symptoms over the past two weeks were measured using the Short Moods and Feelings Questionnaire (SMFQ) [39], a 13-item self-report scale designed for youth aged 8–18 years. The SMFQ has demonstrated strong psychometric properties in previous studies [39–41]. Each item was rated on a three-point scale: 0 (“not true”), 1 (“sometimes true”), and 2 (“true”), with higher scores indicating more depressive symptoms. In the current sample, internal consistency was strong (Cronbach *α* = 0.91).

#### Anxiety symptoms

Anxiety symptoms during the past two months were assessed using the five-item Screen for Child Anxiety Related Emotional Disorders (SCARED) [42, 43]. This is a self-report scale for youth aged 8–18 years. Items were rated on a three-point scale: 0 (“not or hardly ever true”), 1 (“somewhat or sometimes true”) or 2 (“very or often true”), with higher scores indicating more anxiety symptoms. The 5-item SCARED is considered promising as a brief screening inventory for anxiety disorders [43]. Internal consistency was acceptable (Cronbach *α* = 0.71).

#### Gender

Gender was assessed with the question: “Are you a boy or a girl?” with response options “Boy” and “Girl”. A follow-up question asked: “Do you identify as something other than boy or girl, or were you born with a different sex than the gender you indicated here in the form?”. Analyses categorized gender by responses to the first question (‘boy’/‘girl’) only.

### Statistical analyses

Descriptive analyses were conducted using IBM SPSS Statistics Version 29. Gender differences in sexual harassment victimization were assessed using Chi-Square Goodness of Fit Tests. To examine how sexual harassment victimization by girls and boys predicted depressive and anxiety symptoms, linear regression analyses were performed separately by gender in Mplus version 8.10 [44]. Because recruitment, consent, and randomization in the broader trial occurred at the school level, analyses accounted for the nesting of data within schools. The nested structure of the data (pupils within schools) was accounted for using the “type = complex” command, with intraclass correlations of 0.018 for depressive symptoms and 0.011 for anxiety symptoms. Non-normality in the outcome variables was addressed using the robust maximum likelihood (MLR) estimator. Missing data were handled using Full information Likelihood (FIML) [45], and means of the independent variables were included to retain cases with partial data in the analyses.

## Results

Boys were more frequently identified as perpetrators of sexual harassment than girls. Across genders, harassment by boys was most common. Within-gender comparisons showed that girls were significantly more likely to report harassment by boys (48.5%) than by girls (28.8%), *X*^*2*^ (1, *N* = 1038) = 177.37, *p* < .001. Similarly, boys reported more harassment from boys (29.2%) than from girls (14.7%), *X*^*2*^ (1, *N* = 950) = 194.09, *p* < .001. Between-gender comparisons revealed that girls were significantly more likely than boys to report being harassed by boys (48.5% vs. 29.2%, *X*^*2*^ (1, *N* = 1988) = 77.50, *p* < .001, as well as by other girls (28.8% vs. 14.7%, *X*^*2*^ (1, *N* = 1988) = 56.06, *p* = .01). Overall, 53.9% of girls and 32.3% of boys reported at least one incident of sexual harassment in the past two months.

Linear regression analyses examined how sexual harassment by boys and girls predicted depressive and anxiety symptoms (Table 2). Among boys, harassment by boys was significantly associated with both depressive (β = 0.21, *p* < .001) and anxiety symptoms (β = 0.11, *p* < .05), whereas harassment by girls showed no significant association.Among girls, harassment by boys predicted higher levels of depressive (β = 0.26, *p* < .001) and anxiety symptoms (β = 0.11, *p* < .01) while harassment by girls also showed weaker but significant links to both depressive (β = 0.15, *p* < .01) and anxiety symptoms (β = 0.12, *p* < .05).

**Table 2:** Linear regression analyses of sexual harassment victimization as predictor of depressive and anxiety symptoms

	Unstandardized beta (b)	S.E.	b/S.E.	Standardized beta
Predictors of Depressive symptoms				
BOYS (*n* = 921)				
Harassed by boys	0.82	0.15	5.44***	0.21
Harassed by girls	0.04	0.17	0.25	0.01
GIRLS (*n* = 1028)				
Harassed by boys	0.87	0.16	5.27***	0.26
Harassed by girls	0.72	0.25	2.87**	0.15
Predictors of Anxiety Symptoms				
BOYS (*n* = 921)				
Harassed by boys	0.14	0.07	2.08*	0.11
Harassed by girls	0.12	0.09	1.38	0.08
GIRLS (*n* = 1028)				
Harassed by boys	0.12	0.04	2.80**	0.11
Harassed by girls	0.18	0.08	2.42*	0.12

*SE* standard error; *b/SE* t-statistic; *= *p* < .05, **= *p* < .01, ***= *p* < .001

## Discussion

This study examined how the gender of the perpetrator related to the prevalence of sexual harassment as well as its associations with depressive and anxiety symptoms among adolescents aged 13 to 15. It contributes to a more comprehensive understanding of sexual harassment among adolescents by highlighting the role of perpetrator gender in shaping both the occurrence and the mental health consequences of such experiences. Both girls and boys most often reported boys as perpetrators. Furthermore, harassment by boys was more consistently linked with elevated symptoms of depression and anxiety symptoms in the victims, across genders. In contrast, harassment by girls was linked to these symptoms only in girls. This aligns with earlier research showing that sexual harassment is more often male-perpetrated and disproportionately affects girls [5, 8]. The results are also consistent with Felix and McMahon’s [27] findings that harassment by boys, but not girls, is associated with increased psychological distress in both boys and girls. Harassment by boys may carry a greater psychological burden because it is rooted in power structures and often normalized in peer culture, making it harder to resist or speak out [13–15, 46]. The findings also challenge assumptions in the developmental perspective, which links harassment primarily to increased mixed-gender interaction and awkward sexual exploration during puberty [17, 47]. While the assumptions from the developmental perspective may explain some of the cross-gender sexual harassment, the frequency and emotional toll of same-gender harassment, particularly among boys, point to additional mechanisms such as peer dominance and enforcement of masculinity norms [30].

Although less frequently reported, we found that some boys were sexually harassed by girls. This may involve reactive aggression among girls, perhaps being a defensive or retaliatory response to prior harassment or perceived threat from boys. This pattern would be different that proactive aggression assumed to be motivated by the anticipation of a reward, such as attempts to gain attention [48]. Research indicates that older children and adolescents who engage in sexual harassment are often themselves more likely to have been harassed [49, 50], supporting the assumption of potential cycles of victimization and aggression. Similarly, a study on adolescent dating violence found that girls who reported perpetrating violence against male partners frequently cited self-defense as the primary motive for this behaviour [51]. Together, these findings suggest that for some girls, harassment of boys may stem from earlier victimization or attempts to regain a sense of control within gendered peer hierarchies rather than dominance or flirtation.

While reactive aggression may also play a role in sexual harassment among girls, empirical research on girl-to-girl sexual harassment remains limited. In a U.S. study, more than half of the girls reported being most upset by harassment from boys, while one-third were most upset when the perpetrator was another girl [16]. This is consistent with the present findings, indicating that being harassed by girls, also affected girls’ mental health. In this study, girls also engaged in indirect forms of sexual harassment, such as spreading sexual rumours. Prior research suggests that such behaviours often serve as intrasexual competition strategies, targeting peers perceived as sexually available or attractive [52]. Girls who harass other girls may also act within a social system that rewards conformity and status, rather than out of malice or a desire for domination [53]. Verbal harassment was the most common form of harassment among girls in the current study. Girls may use sexualized labels such as “slut” or “whore” as a form of social control to police other girls’ behaviour [54]. Qualitative research supports this interpretation, showing that gossip, rumour spreading, and verbal shaming function as ways to negotiate femininity and social standings within peer groups by defining what counts as acceptable female sexuality [55]. Slut-shaming among girls has also been linked to poorer mental health outcomes, including higher levels of depressive symptoms and suicidal thoughts [56]. Together, these findings suggest that girl-to-girl sexual harassment can reflect social competition, status signalling, and the internalization of gendered norms about sexuality and the body, mechanisms that may help explain why girls targeted by other girls report emotional distress.

The observed associations in this study align with previous longitudinal research suggesting that being bullied, particularly by boys, may contribute to worsening mental health among victims of both genders [57]. At the same time, adolescents with higher levels of depressive or anxiety symptoms may be more vulnerable to being targets of sexual harassment. Future longitudinal studies are needed to better understand these pathways.

The findings indicate that mechanisms emphasized by the MeToo movement, such as gendered power, male perpetration, and emotional harm, may also be present among young adolescents in secondary schools. Yet these dynamics have so far received less public and scholarly attention in the school context [58]. In Norway, schools are legally obligated to prevent and respond to sexual harassment [7]. The fact that nearly half of the girls reported recent harassment by boys, despite these legal protections, points to a significant gap between policy and practice. Schools remain one of the most supervised environments in adolescents’ lives, and yet harassment continues at striking levels.

### Limitations

Several limitations should be considered when interpreting these findings. First, the parental consent rate was 53.3%, which raises the possibility of selection bias. It remains unclear whether pupils whose parents declined consent differed systematically in their experiences of sexual harassment or mental health. Information on reasons for individual nonresponse among consented pupils was not available, limiting the ability to assess potential differences between respondents and nonrespondents. Unfortunately, it was not possible to compare respondents and nonrespondents, as no individual-level information (such as gender, grade, or other demographic characteristics) was available for pupils without parental consent, in accordance with ethical and data protection regulations in Norway. The overall response rate of 36.8% may therefore have introduced selection bias and restricted the generalizability of the findings. Future research should aim for higher response rates or evaluate potential differences between respondents and non-respondents to strengthen representativeness. Second, the cross-sectional design does not allow for causal inference. Although sexual harassment was associated with depressive and anxiety symptoms, it cannot be determined whether sexual harassment contributed to these symptoms, whether adolescents experiencing psychological distress were more likely to be targeted, or whether both mechanisms operated simultaneously. Longitudinal research is needed to clarify these temporal and causal pathways. Third, this study focused primarily on gender, while other important factors, such as ethnicity and socioeconomic background, were not included. The absence of data on participants’ socioeconomic background limits the ability to assess how such factors may have influenced participation or shaped experiences of sexual harassment and related mental health outcomes. Future research should examine how intersecting social characteristics contribute to the occurrence and psychological consequences of sexual harassment, to provide a more comprehensive and inclusive understanding. Finally, the sexual harassment scale included a broad range of behaviours, including verbal, nonverbal, physical, and digital forms. While this breadth is consistent with standard conceptualizations, it may also encompass heterogeneous experiences that vary in severity and impact.

### Conclusion

The high prevalence and potential mental health consequences of sexual harassment among adolescents, particularly boys’ harassment of both girls and boys, highlight the urgent need for preventive efforts in secondary schools. These findings emphasize the importance of paying closer attention to the experiences of young adolescents, who appear particularly vulnerable to male-perpetrated harassment. Sexual harassment may be a contributing factor to the rise in adolescent mental health problems observed during the 21st century [59].Thus, sexual harassment should be routinely addressed in clinical assessments, similar to bullying. Public authorities and school administrators must take these issues seriously, especially given their legal responsibility to prevent and respond to sexual harassment in educational settings [7]. Greater collaboration between schools and child and adolescent mental health services is essential to ensure that incidents of sexual harassment are recognized, appropriately managed, and followed by coordinated support.

## Data Availability

Restrictions apply to the availability of these data, and are thus not publicly available.
